# Multimodal objective assessment of a porcine limbal stem cell deficiency model for corneal therapy research

**DOI:** 10.1038/s41598-025-32842-w

**Published:** 2025-12-20

**Authors:** Piotr Lewandowski, Aleksandra Kowalik, Krzysztof Pietryga, Katarzyna Jesse, Joanna Zembala-John, Rafał Jakub Bułdak, Adam Konka, Edyta Reichman–Warmusz, Romuald Wojnicz, Edward Wylęgała, Dariusz Dobrowolski

**Affiliations:** 1 Department of Histology and Cell Pathology, Faculty of Medical Sciences in Zabrze, Medical University of Silesia in Katowice, Zabrze, H. Jordana 19, 41-808 Zabrze, Poland; 2 Silesian Park of Medical Technology Kardio-Med Silesia, M. Curie-Skłodowskiej 10c, 41-800 Zabrze, Poland; 3 Acellmed Ltd., M. Curie-Skłodowskiej 10c, 41-800 Zabrze, Poland; 4 Department of Environmental Medicine and Epidemiology, Faculty of Medical Sciences in Zabrze, Medical University of Silesia in Katowice, H. Jordana 19, 41-808 Zabrze, Poland; 5 Department of Clinical Biochemistry and Laboratory Diagnostics, Institute of Medical Sciences, University of Opole, Oleska 48, 45-052 Opole, Poland; 6 The Centrum e-Zdrowia (e-Health Centre), Ministry of Health of the Republic of Poland, Warsaw, Poland; 7 Chair and Clinical Department of Ophthalmology, Faculty of Medical Sciences in Zabrze, Medical University of Silesia in Katowice, Panewnicka 65, 40-760 Katowice, Poland; 8 Vice-Rector for Development and Technology Transfer, Medical University of Silesia, Poniatowskiego 15, 40-055 Katowice, Poland; 9 Department of Ophthalmology, Trauma Center, St. Barbara Hospital, Medyków Square 1, 41-200 Sosnowiec, Poland

**Keywords:** Porcine cornea, Limbal stem cell deficiency, Optical coherence tomography, Digital pathology, Regenerative medicine, Advanced therapy medicinal product, Diseases, Medical research, Stem cells

## Abstract

Reliable preclinical models that recapitulate human limbal stem cell deficiency (LSCD) and provide objective outcome measures are essential for advancing corneal cell therapies. We induced LSCD in four porcine eyes by total corneal epithelial debridement, tracked healing with serial anterior-segment optical coherence tomography (OCT) (preinjury, 0, 5–11, 17, 23, 28 days), and quantified histological changes via computer-assisted digital pathology. OCT confirmed complete epithelial removal and a reproducible stromal edema peak on days 7–9, followed by incomplete regression by day 28. Histology revealed epithelial hyperplasia, keratinization, inflammatory infiltration and neovascularization, whereas cytokeratin-3 staining revealed patchy loss of the corneal phenotype. The automated analysis revealed significant increases in epithelial roughness (*p* = 0.049), thickness heterogeneity (*p* = 0.0001), and stromal cellularity (*p* = 0.0179) in experimental eyes compared with control eyes. These multimodal, bias-free metrics clearly distinguish healthy corneas from LSCD corneas and provide quantifiable end points for preclinical testing of limbal epithelial, iPSC-derived or mesenchymal stromal cell advanced therapy medicinal products. All the data and analysis codes are provided in the Supplementary Information.

**Supplementary Information:**

The online version contains supplementary material available at 10.1038/s41598-025-32842-w.

## Introduction

Corneal injuries are a common reason for visits to emergency departments. In most cases, abrasions involve only a small, superficial area of the epithelium, which typically regenerates within 24–48 h. However, in more severe cases, these injuries can lead to corneal remodeling, including scarring and vascularization, ultimately resulting in blindness^1^. In such cases, the standard treatment is corneal transplantation^2^. In eyes where the regenerative potential of the epithelium is inefficient and where symptoms of epithelial stem cell deficiency are present, accompanied by secondary invasion of the vascularized conjunctival tissue, reconstructive treatment, consisting of delivery of stem cells to the corneal epithelium, is necessary. In monocular injuries, the contralateral eye becomes a perfect donor of the limbus, which is the source of epithelial cells. Another extensively researched technique involves autologous limbal stem cell transplantation^3,4^.

The development of novel corneal therapies necessitates the use of appropriate animal models. Our current understanding of corneal regeneration is predominantly based on studies conducted in rabbits, as confirmed by a recent systematic review of 105 preclinical studies on limbal stem cell deficiency (LSCD), which revealed that the vast majority of these studies involved rabbits and that no studies involved pigs^5^. In addition, a more recent critical narrative review identified no reported LSCD porcine models^6^. Owing to their eye size, rabbits are commonly used in ophthalmic research^5^, and their corneas are architecturally similar to those of humans. Nevertheless, important differences, such as less stromal neovascularization, fewer goblet cells, and a lower inflammatory response following ocular insult, limit the direct translation of findings^5^. Similarly, rodent corneas lack the Palisades of Vogt, which in humans provide crucial protection for limbal stem cells, thereby increasing the susceptibility of murine limbal stem cells to external insults^5^. While cats and nonhuman primates are more comparable to humans, the smaller size of cats’ eyes and the high cost and limited availability of nonhuman primates pose considerable challenges^6^. Given these limitations, pigs are promising alternative models. Pigs offer a unique compromise, with eyes similar in size and anatomy to those of human eyes, along with comparable mechanisms of corneal regeneration^7^.

Assessment of corneal regeneration remains a significant challenge in both preclinical and clinical settings. According to the aforementioned systematic review, most studies rely on conventional clinical observations—such as fluorescein staining and impression cytology—often performed at only a few distinct time points, thereby limiting the consistency of longitudinal data^5^. While these approaches help identify epithelial defects and changes in ocular surface morphology, they often lack the reproducibility and sensitivity required for precise monitoring of corneal healing. More recently, optical coherence tomography (OCT) has emerged as a promising, noninvasive tool for assessing LSCD, enabling real-time, cross-sectional visualization of the corneal microarchitecture^8^. This technique facilitates repeated and quantitative evaluations of corneal regeneration, potentially improving the reliability of clinical assessment and guiding more informed therapeutic interventions.

Several studies highlighted in the literature have employed similar approaches for evaluating corneal regeneration, predominantly relying on human-based grading or observation. In mouse models, researchers often combine slit-lamp examinations and fluorescein scoring with immunofluorescence staining to visualize neovascularization, inflammation, and epithelial markers, with assessments performed by human observers^9,10^. In rabbit models, clinical evaluations during follow-up are common and are occasionally supported by pathological examinations^11^. Some investigators have incorporated quantitative measures into their protocols; for example, one study employed ImageJ software to analyze clinical photographs while also using a predefined scale for histological evaluation^12^, and another involved a histopathologist to quantify distinct cell phenotypes via immunofluorescence^13^. Nevertheless, many experiments continue to rely on observer-dependent scoring systems for both clinical and histological endpoints^14^, underscoring a broader trend toward subjective or semiquantitative assessments. Notably, the adoption of ImageJ in clinical assessment represents a positive shift toward more objective, computer-assisted analyses, suggesting that future studies can build on this approach to increase repeatability and minimize observer bias.

Recent studies have characterized the porcine ocular surface in remarkable detail, revealing the quality of both classical histology and immunohistochemical staining, which is possible with porcine tissues^15^. Classical histology remains indispensable for capturing the morphological details critical to diagnosing and understanding corneal regeneration; however, its reliability can be undermined by observer dependence. Even the most refined histological grading scales and expert pathologists are still susceptible to subjective interpretation, leading to reduced consistency and repeatability. Current techniques in immunohistochemical and molecular analyses have advanced our insight into the corneal cell phenotype, lineage markers, and signaling pathways. Nevertheless, these methods introduce further technical complexities. Importantly, molecular changes do not necessarily equate to functional restoration of the cornea. While immunohistochemical data yield valuable information on tissue composition and cellular processes, they must be integrated with classical histological findings to provide a more comprehensive picture of corneal healing. As a complementary approach, digital pathology and automated image analysis can help mitigate observer bias, enhancing both the consistency and objectivity of tissue assessments.

Therefore, the primary goal of this study was to develop and validate the first porcine model of LSCD while integrating two robust and objective methodologies for assessing corneal regeneration. First, OCT was performed at multiple time points to provide real-time, quantitative measurements of corneal thickness. Second, a digital pathology workflow was introduced, defining critical morphological parameters that can be automatically calculated to reduce observer dependence. Altogether, this approach aims to offer a repeatable and easy-to-use evaluation strategy for LSCD that is largely independent of human bias, thereby streamlining and improving the reliability of corneal research.

## Results

### Corneal damage and regeneration

**Fig. 1:**
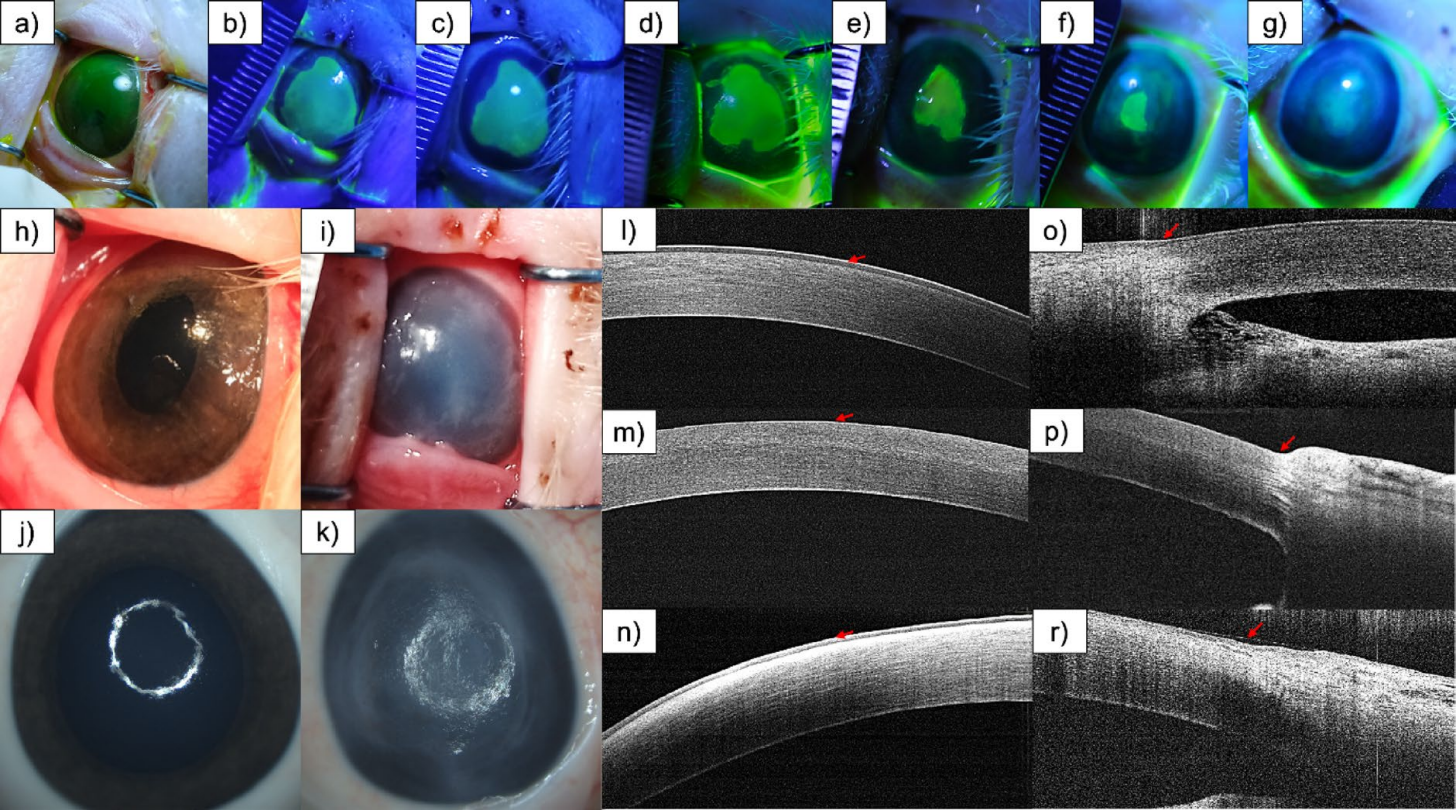
Representative images of damaged and healed corneas; Fluorescein-stained corneas illustrating the progression of healing: immediately after epithelial damage (**a**) and at days 5 (**b**), 7 (**c**), 10 (**d**), 17 (**e**), 23 (**f**), and after euthanasia (**g**). Gross images of the corneas before damage and after healing were taken during the procedure (**h**, **i**) and ex vivo (**j**,** k**). Optical coherence tomography (OCT) images showing the central (**l**–**n**) and limbal (**o**–**r**) regions of the cornea before damage, immediately after the procedure, and following the healing process. Red arrows indicate the epithelial layer: intact epithelium in (**l**,** o**), complete epithelial loss in (**m**,** p**), and re-established epithelium after healing in (n, r).

Postoperative examination with fluorescein solution and blue light confirmed the complete removal of the corneal epithelium (Fig. 1a). The regeneration process was gradual, with epithelial ulceration healing occurring over 28 days (Fig. 1b–g). Gross examination revealed a loss of corneal transparency (visible in Fig. 1h–k ) during the recovery phase. OCT further validated the removal of the corneal epithelium and documented its subsequent regeneration (Fig. 1l–r, with red arrows indicating the location of the epithelium). Representative slit-lamp photographs and quantitative analysis of fluorescein-stained corneal defect area (expressed in mm² and as a percentage of total corneal surface) at serial follow-up time points are provided in Supplementary Figure S1.

**Fig. 2:**
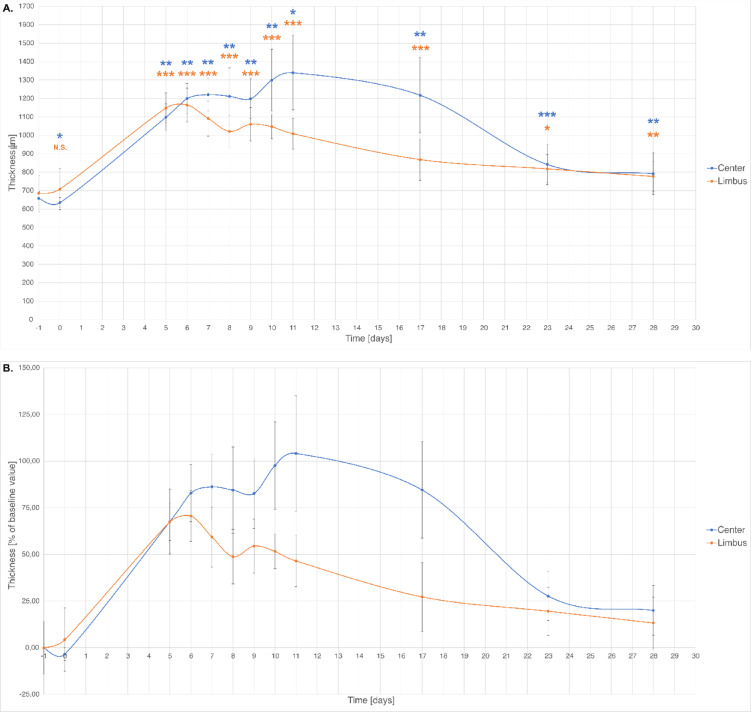
Quantitative analysis of epithelial thickness in the porcine cornea following limbal epithelial removal. (**A**) Absolute epithelial thickness (µm) measured by OCT at each time point in the central and limbal regions. (**B**) Relative changes in epithelial thickness expressed as percentage of baseline (Day-1) values. Data are shown as mean ± SD from four animals. Statistical comparisons were performed using a paired Student’s t-test relative to baseline (Day-1). Statistical significance: *p* < 0.05 (*), *p* < 0.01 (**), *p* < 0.001 (***), n.s. – not significant.

The average thickness measurements revealed significant stromal swelling immediately following the procedure, with the swelling peaking around days 7–9 in the central cornea and slightly earlier in the limbus (Fig. 2). Both regions exhibited a sharp increase in thickness post-procedure, followed by a gradual decline over 28 days. However, the central cornea consistently displayed greater swelling and slower resolution than did the limbus, remaining persistently thicker throughout the experiment. Although stromal thickness progressively decreased over time as part of the regeneration process, it never fully returned to baseline values, and the thickness approached but did not reach the original predamage levels.

## Histological assessment and digital pathology evaluation

**Fig. 3:**
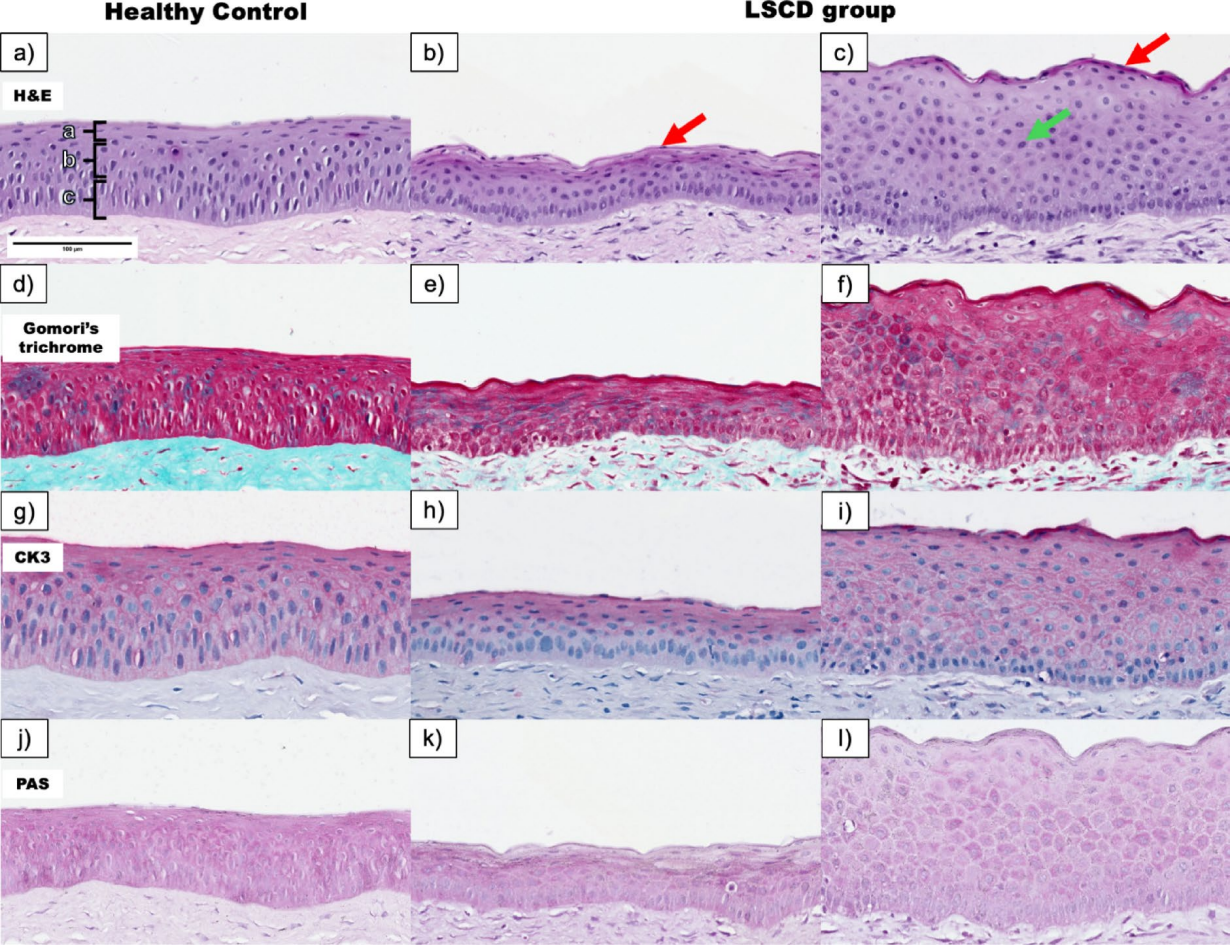
Pathological changes in the central corneal epithelium at Day 28. In the healthy control samples, the epithelium shows a normal thickness with distinct cellular layers, whereas the limbal region has intact crypts and a well-organized collagen architecture (**a**, **d**, **g**, **j**). In contrast, the study group exhibited regions of epithelial thinning, characterized by keratinization of the superficial layer (red arrow), reduced epithelial layers, and proliferation of basal cells (**b**, **e**, **h**, **k**). Areas of epithelial thickening display increased numbers of cellular layers, increased keratinization (red arrow), and altered wing cell morphology (green arrow) (**c**, **f**, **i**, **l**). Brackets in panel (a) indicate the three major epithelial layers: (a) basal cells, (b) wing cells, and (c) superficial cells.

The corneal samples were subjected to microscopic analysis, with a focus on the anterior corneal epithelium, limbal region, and corneal stroma. Undamaged eyes served as comparative controls. Microscopic evaluation revealed significant structural changes in the corneal epithelium of the study group compared with the controls. Although the overall mean epithelial thickness differences between the groups were subtle, pronounced localized areas of thickening and thinning were observed (Fig. 3a–l). Thinning regions (Fig. 3b, e, h, k) were characterized by a reduced number of cellular layers, keratinization of the superficial layer, and dense proliferation of basal cells, suggesting disrupted differentiation and compensatory hyperproliferation.

**Fig. 4:**
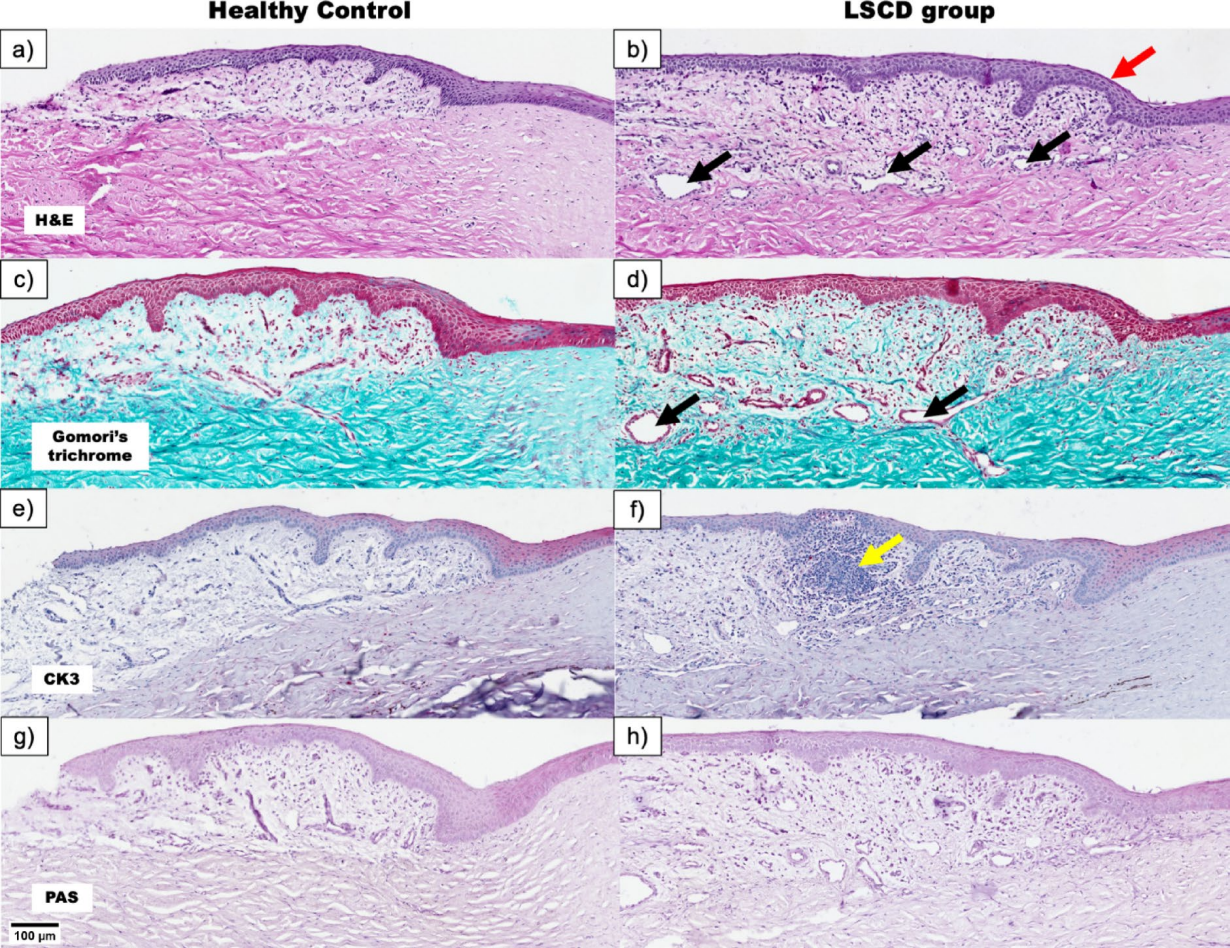
Pathological changes in the corneal limbus at Day 28. The limbal region in the control group (**a**, **c**, **e**, **g**) shows intact crypts and organized stromal collagen. In the study group (**b**, **d**, **f**, **h**), microscopic analysis revealed disrupted crypts, increased keratinization (red arrow), inflammatory infiltrates (yellow arrow), and neovascularization (black arrows), reflecting structural and functional damage.

In contrast, the thickened areas (Fig. 3c, f, i, l) presented an increased number of epithelial layers, an altered morphology of wing cells with a more rounded appearance, and keratinization of the superficial layer, indicative of pathological hyperplasia. CK3 staining revealed apparent differences in cytokeratin expression between the study and control groups. In healthy controls (Figs. 3g and 4e), CK3 was strongly expressed in superficial and wing cells, with reduced expression in basal cells, which was consistent with normal corneal differentiation. However, in the study group (Figs. 3h, i and 4f), CK3 expression was absent in basal cells. It showed patchy and irregular staining in superficial and wing cells, indicating disrupted differentiation and altered epithelial homeostasis.

The limbal region in the study group presented distinct pathological changes, including disorganization of the limbal crypts, which serve as stem cell niches, with the crypts appearing less distinct and disrupted (Fig. 4b, d, f, h) compared to controls (Fig. 4a, c, e, g). Inflammation and increased vascularization were evident, with cellular infiltration and blood vessel proliferation extending into the limbal stroma. Compared with those in the control group, the basement membrane was irregular, and the collagen fibers in the stroma were more disorganized, suggesting that remodeling occurred in response to injury (Fig. 4g, h).

**Fig. 5:**
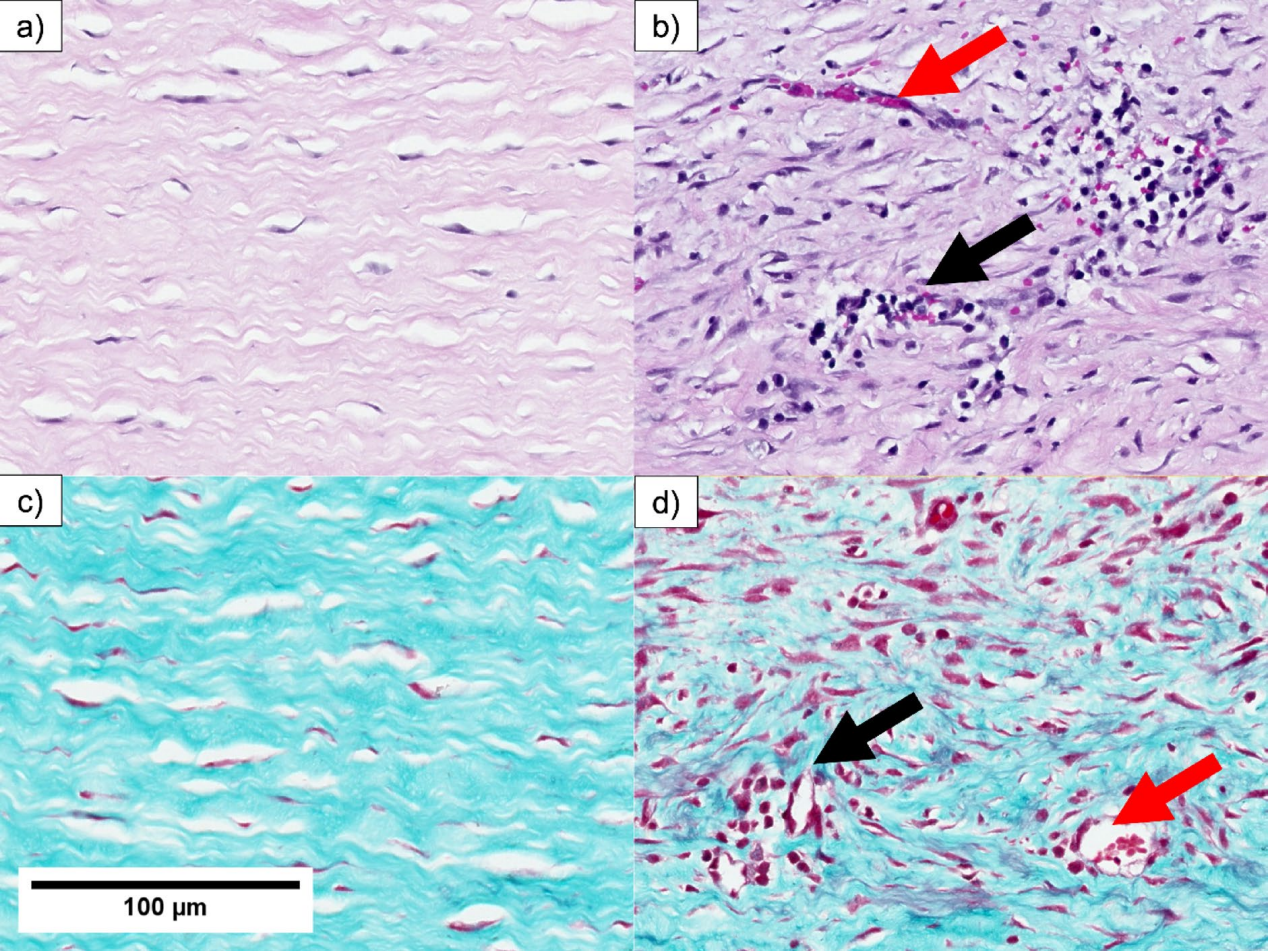
Pathological changes in the corneal stroma of healthy controls (**a**, **c**) and the LSCD model (**b**, **d**) at Day 28. First row—H&E, second row—Gomori’s Trichrome. Inflammatory infiltration—black arrow, vascular invasion—red arrow. The figure additionally shows that the LSCD model leads to a disruption of the lamellar structure of the cornea, which becomes more chaotic and irregular than the organized appearance observed in healthy controls. This disorganization is particularly evident in images (b) and (d), where the normal parallel arrangement of stromal fibers is replaced by a more disordered and heterogeneous structure, accompanied by inflammatory and vascular changes.

Pathological changes in the corneal stroma were also evident in the study group, highlighting a robust remodeling response to injury. Inflammatory infiltration was prominent, with clusters of inflammatory cells disrupting normal stromal organization (Fig. 5b, d). Enlarged blood vessels were observed within the stromal region, indicating vascular invasion. The typical lamellar and parallel arrangement of stromal collagen fibers observed in the control group (Fig. 5a, c) was replaced in the study group by a chaotic and heterogeneous structure (Fig. 5b, d). Regions of edema were visible, reflecting swelling and extracellular matrix remodeling. Masson’s trichrome staining further revealed these structural differences, with an irregular arrangement of collagen fibers and increased cellular infiltration in the study group compared with the control group (Fig. 5c, d).

**Fig. 6:**
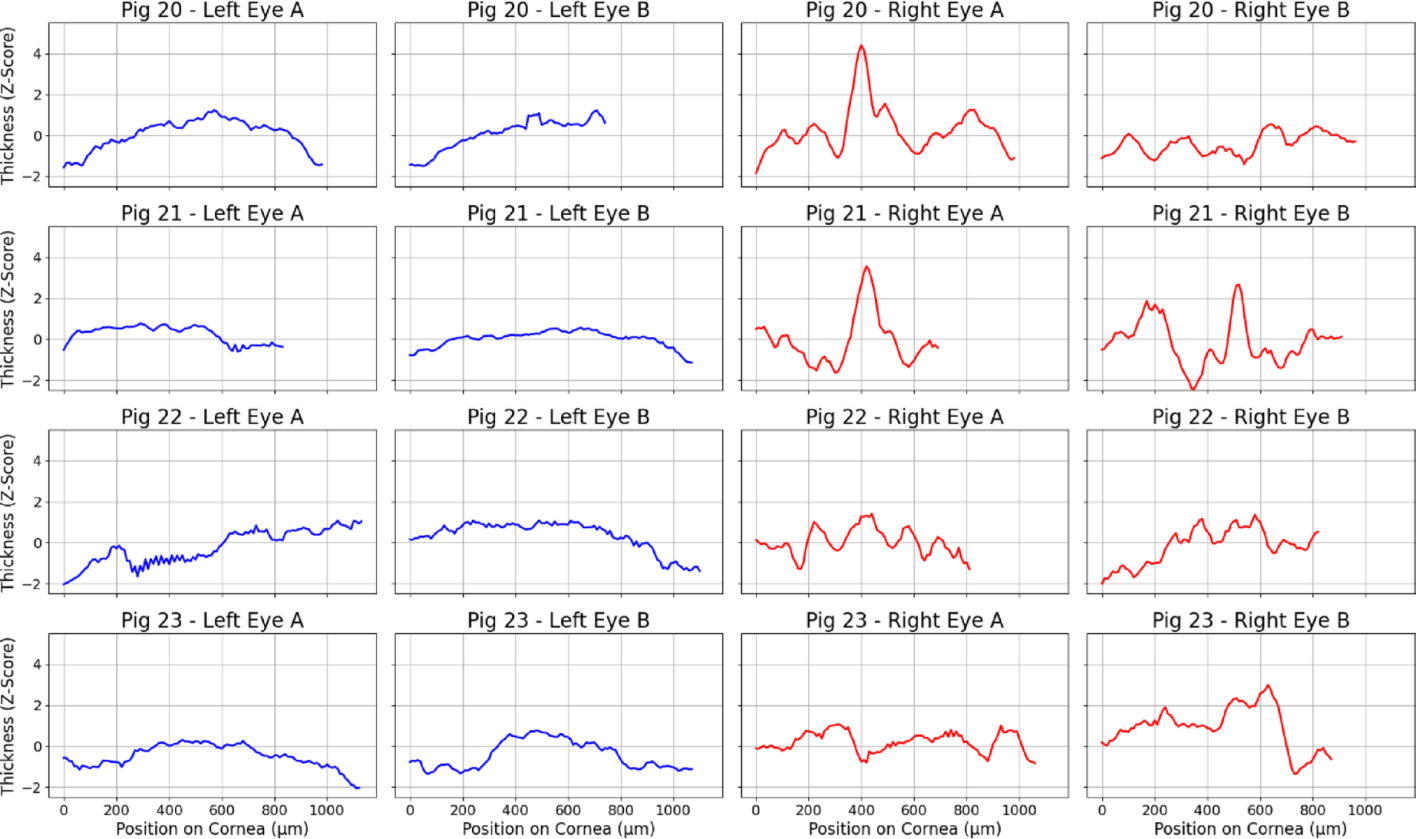
Thickness profiles of the anterior corneal epithelium. The thickness of the epithelium was measured via histological section scans. The profiles illustrate differences between the control group (blue) and the LSCD group (red). In the LSCD group, there was a significant increase in the roughness of the corneal epithelium and extreme thickness values, indicating localized irregularities in response to the injury.

Digital pathology revealed distinct patterns of epithelial thickness variation and cell density distributions in the study group compared with the controls (*p* = 0.0179). Analysis of surface epithelial thickness (*p* = 0.0001) and the maximum height of the profile (R_z_, *p* = 0.04940) revealed significant disruptions in the epithelial surface of damaged corneas, with localized areas of extreme thinning and thickening contributing to irregularity. The other parameters did not significantly differ (max–min difference, arithmetic mean roughness Ra, skewness and standard rolling deviation, and epithelial cell density). The quantitative results are summarized in Table 1, and the epithelial thickness profiles are presented in Fig. 6.

**Table 1:** Results of the digital pathology evaluation. The mean value is ± standard deviation.

Parameter		Study group	Control group	*p* value
Number of samples (n)		4	4	
Corneal thickness	Number of measurements (total)	718	812	
	Number of measurements per eye (avg)	179.50 ± 18.52	203.00 ± 24.29	0.17480^‡^
	Epithelium thickness (avg total)	**80.47 ± 18.39**	**77.36 ± 11.49**	**0.00010** ^**†**^
	Epithelium thickness (avg of avgs)	80.20 ± 6.59	77.67 ± 4.27	0.57160^‡^
	Minimal epithelium thickness (avg)	37.51 ± 13.11	49.02 ± 2.83	0.21180^‡^
	Maximum epithelium thickness (avg)	126.67 ± 21.50	95.67 ± 3.51	0.15430^‡^
	Max-min difference (avg)	78.11 ± 47.37	63.00 ± 33.51	0.50570^‡^
	Arithmetic Mean Roughness (Ra)	0.83 ± 0.19	0.68 ± 0.21	0.18160^‡^
	Maximum Height of Profile (Rz)	**4.39 ± 1.32**	**2.88 ± 0.73**	**0.04940** ^**‡**^
Corneal thickness distribution	Measurements exceeding 1 σ	12.53% ± 10.82	13.18% ± 18.46	0.86530^‡^
	Measurements exceeding 2 σ	3.90% ± 2.57	1.60% ± 3.74	0.08000^‡^
	Measurements exceeding 3 σ	1.25% ± 1.45	0.25% ± 0.57	0.23020^‡^
	Skewness	0.73 ± 0.98	-0.43 ± 0.46	0.08550^‡^
	Rolling Standard Deviation	8.64 ± 2.63	4.36 ± 1.25	0.06860^‡^
Epithelium cells density	Analyzed Area (total) – mm^2^	18.99	9.19	
	Analyzed Area Mean Per eye (avg) – mm^2^	2.54 ± 3.05	1.15 ± 0.08	0.4990^‡^
	Cell density (total) – cells/mm^2^	2205.0	6394.0	
	Cell density (avg) – cells/mm^2^	4271.0 ± 2184.0	6390.0 ± 762.0	0.1683^‡^
Stroma cells density	Analyzed Area (total) – mm^2^	45.84	48.52 ±	
	Analyzed Area Mean Per eye (avg) – mm^2^	5.73 ± 0.78	6.07 1.38	0.6330^‡^
	Cell density (total) – cells/mm^2^	1272.0	548.0	
	Cell density (avg) – cells/mm^2^	**2534.0 ± 662.1**	**1082.0 ± 224.0**	**0.0179** ^**‡**^

† – unpaired T Test, ‡ – paired T Test. Significance are in bold.

## Discussion

In this study, we established a porcine model of LSCD and introduced a more objective assessment of corneal regeneration. Our key findings demonstrate that (1) controlled epithelial debridement with OCT successfully induces LSCD-like pathology; (2) histological and immunohistochemical analyses reveal hallmark changes consistent with impaired corneal repair; and (3) digital pathology parameters—including the maximum height of profile (Rz), epithelial thickness, and stromal cell density—distinguish injured corneas from healthy corneas with minimal observer bias.

Building on the anatomical and physiological similarities between porcine and human corneas, our findings confirm that controlled epithelial debridement in pigs reliably induces an LSCD-like pathology. Notably, we observed histopathological features comparable to those reported in human LSCD, including fibrosis, inflammatory cell infiltration, epithelial hyperplasia, and signs of squamous metaplasia^16^. We did not detect goblet cells in our porcine samples; however, their absence is often associated with squamous metaplasia, as previously documented in human samples^16^. These robust remodeling patterns further validate the porcine model as a valuable platform for translational research on corneal wound healing and LSCD management.

Serial OCT provided real-time, quantitative tracking of corneal changes. After epithelial debridement, we noted pronounced stromal swelling peaking at days 7–9, followed by gradual resolution. Similar edema patterns are observed in rabbits and rodents, but the timelines differ. Interestingly, the stromal thickness did not fully return to baseline by day 28, suggesting ongoing remodeling. Clinically, prolonged swelling may predict corneal haze or scarring, underscoring the importance of extended follow-up in injury models.

Our histopathological and immunohistochemical analyses aligned with well-documented LSCD features—namely, disruptions in limbal stem cell niches, inflammatory infiltration, and aberrant epithelial differentiation patterns. In particular, altered cytokeratin-3 (CK3) expression indicated compromised epithelial stratification, suggesting that the porcine model faithfully replicates key aspects of LSCD pathophysiology observed in humans. This observation is consistent with previous reports demonstrating reduced CK3 expression in the corneal epithelium in a rabbit LSCD model^17^. Furthermore, mature corneal epithelial cells in humans typically express CK3, whereas the limbal region does not^18,19^. Corneal regeneration is believed to proceed through centripetal migration of limbal stem cells toward the site of injury, followed by their transdifferentiation into corneal epithelial cells^20^. In such a scenario, the new epithelium gradually upregulates CK3, although at a lower level than that in uninjured tissue^20^. Interestingly, even in patients with clinically confirmed LSCD, CK3-positive cells have been detected by impression cytology^21^, suggesting that some degree of corneal differentiation may still occur. A similar process in our porcine model could account for the presence of CK3-positive cells despite LSCD-like conditions.

In many corneal studies, subjective or semiquantitative scoring can obscure subtle tissue changes. In contrast, our automated analysis quantified epithelial thickness, surface roughness (Rz), and cell density, providing more precise metrics. From a biological and potential clinical perspective, Ra and Rz reflect epithelial surface irregularity, which is directly linked to corneal smoothness and thus to optical quality and visual symptoms in LSCD and other ocular surface disorders. Epithelial heterogeneity measures, such as skewness and the proportion of values exceeding 1–3 standard deviations, capture focal “hot spots” of thickening or thinning that may correspond to areas of epithelial instability or early conjunctivalization. Likewise, epithelial and stromal cell density quantify inflammatory infiltration and tissue remodeling, paralleling inflammatory cell counts and haze grading used in clinical practice. Although corneal epithelial thickness has proven highly informative, its true diagnostic power emerges only when compared across multiple animals, ensuring robust insights into pathological alterations. Importantly, relatively few studies have focused on anterior corneal epithelial thickness in LSCD patients in humans. Notably, in vivo laser scanning confocal microscopy has allowed the classification of LSCD into early, intermediate, and late phases, with significant epithelial thickening observed primarily in the intermediate and late stages^22,23^. In early LSCD, the mean thickness can remain comparable to that of healthy eyes yet can exhibit a larger standard deviation, suggesting the presence of local “hot spots” of thickening or thinning^23^. Although these findings are derived from human studies, the focal alterations we detected in porcine corneas may reflect a similar process in clinical settings, supporting the notion that such regional “hot spots” could exist in both species and be targeted by regenerative interventions. Finally, cell density reflects inflammatory infiltration, enabling objective monitoring of immune responses and the efficacy of novel therapeutics aimed at reducing inflammation and promoting tissue repair.

Translationally, this model supports the testing of novel cell- and tissue-based therapies (e.g., advanced therapy medicinal products, ATMPs) in a corneal environment that approximates human eyes. Coupling OCT with automated pathology allows a more precise evaluation of ex vivo expanded limbal stem cells or stromal cell injections.

## Study limitations

Despite promising findings, several limitations should be highlighted:

- Small sample size: The study group included four animals, which may have limited the statistical power of certain findings. Further studies with larger cohorts could strengthen the validation of both the model and the measurement techniques.
- Follow-up duration: A 28-day observation period captured initial re-epithelialization; however, corneal remodeling often lasts a few months. Longer-term studies could provide a more comprehensive understanding of scarring and neovascularization.
- Vascular and conjunctival changes: neovascularization and conjunctivalization were not systematically quantified in this study. Preliminary attempts revealed high variability and asymmetric vessel ingrowth, which could result in biased evaluation depending on the corneal section plane. Moreover, reliable identification of vascular and conjunctival epithelial markers would require reproducible immunohistochemical (IHC) staining, which proved technically challenging in our preliminary trials due to tissue detachment and uneven staining. Development of a robust IHC protocol for corneal tissues remains an important direction for future research. Representative examples demonstrating the variability and visualization challenges associated with neovascularization in the porcine cornea are shown in Supplementary Figure S2.
- Mechanistic insights: Further research is necessary to elucidate the processes underlying localized epithelial thinning and thickening—particularly in “hot spot” areas with extreme deviations. Understanding these phenomena is critical for guiding new regenerative strategies aimed at normalizing corneal architecture.
- Lack of functional correlation: Although our methodology provides objective morphological and quantitative metrics, we did not directly correlate these findings with functional outcomes such as corneal transparency, optical performance, visual acuity, or biomechanical properties. These parameters are essential for understanding the true impact of tissue remodeling on vision and are critical for translational relevance. Future studies will integrate functional endpoints, including in vivo confocal microscopy, optical transmission measurements, biomechanical testing, and behavioral or optical performance assessments, to link morphological features with clinically meaningful recovery.

## Future directions

Further optimization of this porcine LSCD model may involve exploring the severity of different injuries, evaluating alternative imaging methods, such as in vivo confocal microscopy, and extending the observation window for a more comprehensive understanding of chronic remodeling. Basic research is also essential to clarify the transition from molecular and morphological changes to functional recovery—an understanding that could refine therapeutic approaches. Additionally, testing cell-based therapies or scaffolds in this model could offer critical translational insights before advancing to clinical trials, particularly considering regulatory requirements for robust preclinical evidence.

## Conclusion

This study provides a reproducible and objective porcine LSCD model by integrating repeated OCT imaging and digital pathology. By minimizing observer bias and enabling precise, cross-sectional assessments of corneal changes, this approach offers a robust platform for evaluating regenerative interventions. OCT provides high-resolution, longitudinal monitoring to verify model quality, whereas digital pathology facilitates reliable quantification of epithelial disruption and inflammatory processes. Ultimately, this integrated strategy helps bridge the gap between preclinical findings and clinical applications, advancing corneal regeneration research toward more effective therapies and improved patient outcomes.

## Methods

### Animals

The experiment was performed on four domestic pigs (*Sus scrofa domesticus)* obtained from a local breeder. The animals were housed and managed in compliance with the procedures in force at the facility, the study protocol, and the approval of the Local Ethics Committee of the Medical University of Silesia in Katowice (Resolution No. 19/2022). All procedures complied with the ARRIVE 2.0 guidelines^24^, and the checklist is available in Supplementary Data S3. Prior to the commencement of the experiments, the animals were quarantined for three weeks. Each pig’s right eye was used as the LSCD model, whereas the left eye served as the control.

### Corneal damage procedure

Corneal damage was induced by an experienced ophthalmologist under strict aseptic conditions. All participants wore disposable protective clothing, and the lead operator followed the Ayliffe method for hand hygiene, using sterile gloves and gowns. Sterile surgical draping was applied to isolate the surgical field and minimize the risk of contamination.

Before the procedure, the animals were thoroughly cleaned to reduce the risk of contamination from skin microorganisms or fecal matter. Each animal was premedicated with a combination of ketamine (22 mg/kg) and xylazine (2 mg/kg) to induce sedation, followed by intravenous anesthesia using propofol (2–4 mL/10 kg) and fentanyl (0.05 mg/kg/min). Once sedated, the animals were intubated to ensure airway patency during the procedure. Monitoring equipment, including electrodes, was attached to track vital signs such as heart rate, respiration, body temperature, and oxygen saturation.

The procedure aimed to simulate the condition of limbal stem cell deficiency (LSCD) by removing the corneal epithelium. OCT imaging was performed before epithelium removal to document the baseline corneal structure. A 20% ethanol solution prepared with 0.9% saline was applied to the corneal surface for 60 s to soften the epithelium. The eye was then thoroughly rinsed with 0.9% saline for 15–20 s to remove the ethanol and discoloration. The softened epithelium was carefully removed using curved Vannas scissors to cut the conjunctiva at the limbus. Subsequently, approximately 2 mm of corneal epithelium was excised proximally from the incision line, ensuring complete removal of vessels emerging from the limbus. The remaining limbal epithelium was gently removed using a crescent or hockey knife to induce limbal stem cell deficiency. The effectiveness of epithelial removal was verified via a fluorescein test, which involved the application of a 1% fluorescein solution and examination of the corneal surface under UV light. Uniform yellow‒green fluorescence indicated proper removal, whereas areas with residual epithelium remained uncolored. OCT imaging was repeated after epithelium removal to confirm the changes in corneal structure.

Following epithelial removal, partial tarsorrhaphy was performed by suturing the upper and lower eyelids with approximately three single stitches to protect the eye. After the procedure, the animals received three doses of metamizole (30 mg/kg) every 12 h and dexamethasone (0.02–0.06 mg/kg) every 48 h. The animals were monitored postoperatively for signs of discomfort or complications.

### Optical coherence tomography

OCT was performed via the iVue® 80 (OptoVue) apparatus at several time points: before the procedure and on days 0, 5, 6, 7, 8, 9, 10, 11, 17, 23, and 28 after corneal damage. For each examination, the animals were premedicated with a mixture of ketamine and xylazine, followed by intravenous anesthesia maintained with propofol. An intravenous cannula was inserted into the marginal ear vein for drug administration, and the procedure was conducted under continuous monitoring with a cardio monitor to track ECG, oxygen saturation, and core body temperature.

To ensure proper imaging, an eyelid speculum was applied, and the corneal surface was anesthetized with topical proxymetacaine (5 mg/mL, 1–2 drops). Corneal thickness measurements were taken in both the central and limbal regions of the cornea. Following corneal damage, on days 5, 7, 10, 17, and 23 and post euthanasia, the corneas were stained with a 1% fluorescein solution, and images were captured under blue light via a dedicated camera. This approach allows the assessment of epithelial healing and structural integrity.

After the examination, the animals were allowed to recover naturally from anesthesia under observation to ensure their safety and well-being.

### Gross examination, tissue processing, and histochemical analysis

After 28 days, euthanasia was performed via intravenous administration of xylazine (2 mg/kg), ketamine (22 mg/kg), propofol (2–4 ml/10 kg), and pentobarbital (140 mg/kg). Then, the corneas were rinsed with standard saline solution, and the eyes were enucleated. High-resolution images of the corneas were captured via reflected light microscopy (Zeiss AxioZoom). The corneas were then carefully excised with a razor blade, leaving an approximately 1 mm margin of the sclera to preserve tissue integrity.

The iris and lens were subsequently removed, and each cornea was divided into four equal strips. These strips were meticulously labeled as two medial and two lateral parts and then placed in individual histopathological cassettes for further processing. Fresh tissue was maintained in ice-cold phosphate-buffered saline (PBS) before being fixed in a tissue processor. The fixation and dehydration processes involved immersion in neutral buffered formalin, graded alcohols, and xylene, followed by infiltration with molten paraffin. The tissues were then embedded in high-melt paraffin blocks.

The paraffin-embedded corneas were serially sectioned into 5 μm thick slices and stained with hematoxylin and eosin (H&E), Gomori’s trichrome, and periodic acid–Schiff (PAS) stains for histological analysis.

### Immunohistochemical staining

For immunohistochemical (IHC) staining, the slides were mounted onto Superfrost Plus Gold slides (Epredia) and dried overnight at 60 °C to ensure proper adhesion. Antigen retrieval was performed in a microwave oven with citrate-based antigen unmasking solution (Vector H-3300) following the manufacturer’s protocol. The slides were then incubated overnight at 4 °C with a primary antibody against cytokeratin 3 (Invitrogen MA1-5673) at a 1:300 dilution. Detection was achieved via the ImmPRESS-AP Horse Anti-Mouse IgG Polymer Detection Kit (Vector MP-5402), which uses an alkaline phosphatase-based system. A hematoxylin counterstain was applied to visualize the tissue structures.

### Digital pathology and analysis

All slides were digitized via an Ocus scanner (Grundium) equipped with a 20× UPlanXApo objective (Evident) for high-resolution imaging. Quantitative analyses were conducted via QuPath software to measure epithelial thickness and cellularity, which was defined as the number of cells per square micrometer (µm²). Data from the analyses were exported as .csv files for further processing in Python 3.12.2. Surface irregularity metrics were computed in Python using NumPy, Pandas, and SciPy. Skewness of the epithelial thickness distribution was calculated for each pig using the scipy.stats.skew function, representing the asymmetry of local thickness variations. Local epithelial variability was quantified using a rolling standard deviation, computed in a moving window of five consecutive measurements along the linearized epithelial contour. Surface roughness parameters (Ra and Rz) were derived from epithelial thickness profiles standardized as z-scores. The arithmetic mean roughness (Ra) was calculated as the mean of the absolute deviations of standardized epithelial thickness from its mean value along the scanned corneal profile. The maximum height of the surface profile (Rz) was defined as the difference between the maximum and minimum standardized thickness within each profile, reflecting the amplitude of local surface irregularities. Raw .csv file is available as Supplementary Data S4.

The Python script used standardized epithelial thickness data by calculating z scores for each pig to account for individual variability. Smoothing was applied to the thickness profiles via a moving average. Separate profiles were generated for the right and left eyes (slides A and B), and combined charts were created to compare the profiles across samples. Visualizations and statistical plots were created via Matplotlib version 3.9.2. The code for generating thickness profiles is available in Supplementary Data S5.

### LLM usage in manuscript preparation

During the preparation of this work, the author(s) used GPT 4.0 and GPT o3 to improve language and readability. After using this tool/service, the author(s) reviewed and edited the content as needed and take(s) full responsibility for the content of the published article.

## Supplementary Information

Below is the link to the electronic supplementary material.

Supplementary Material 1

Supplementary Material 2

Supplementary Material 3

Supplementary Material 4

Supplementary Material 5

## Data Availability

All raw epithelial-thickness data (csv) and analysis code (Python) are provided as supplementary data.
