# Supplementary material for: Multimodal objective assessment of a porcine limbal stem cell deficiency model for corneal therapy research
Source: Sci Rep. 2025 Dec 20;16:2982. doi: 10.1038/s41598-025-32842-w (PMC12830607; doi:10.1038/s41598-025-32842-w)

Figure S1. Representative slit-lamp photographs and quantitative analysis of fluorescein-stained corneal defect area during follow-up.
(A) Serial slit-lamp photographs at follow-up time points (Day 5, 7, 11, 17, 23, 28) demonstrating the progressive reduction of epithelial defect size as visualized via fluorescein staining in all animals. Day 28 – image was taken after euthanasia.
(B) Quantitative measurement of defect area in square millimeters (mm^2) based on segmentation of fluorescein-positive regions.
(C) Relative defect area expressed as a percentage of the total corneal surface.

(A)

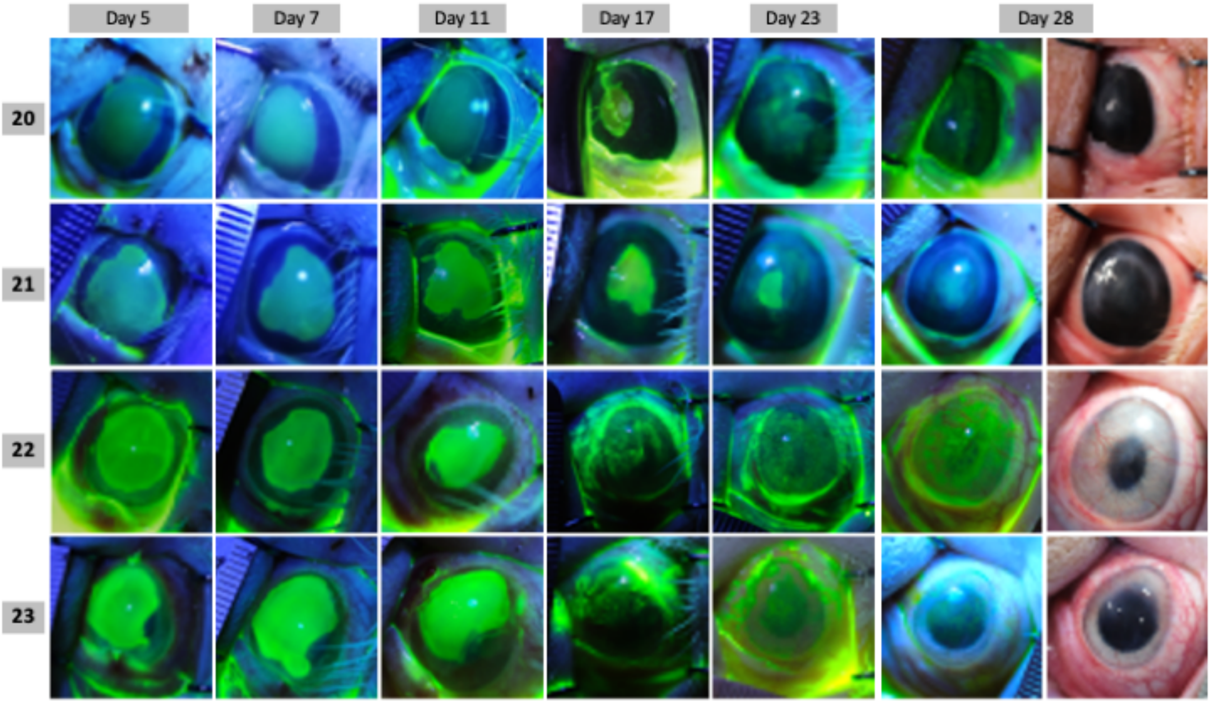

(B)

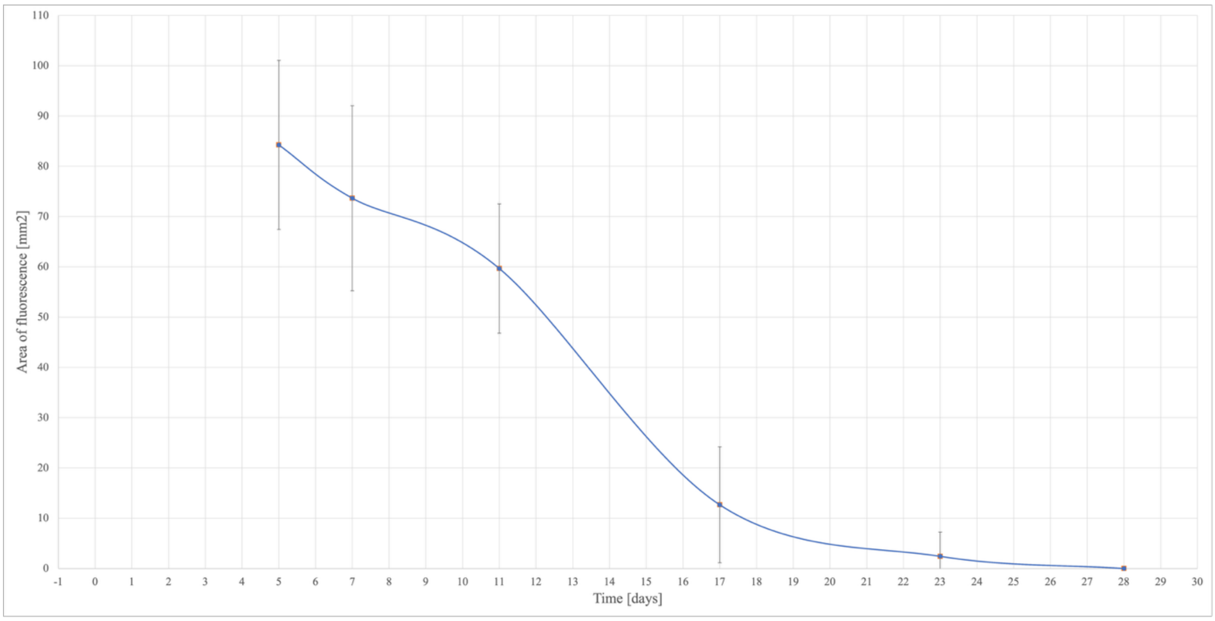

(C)

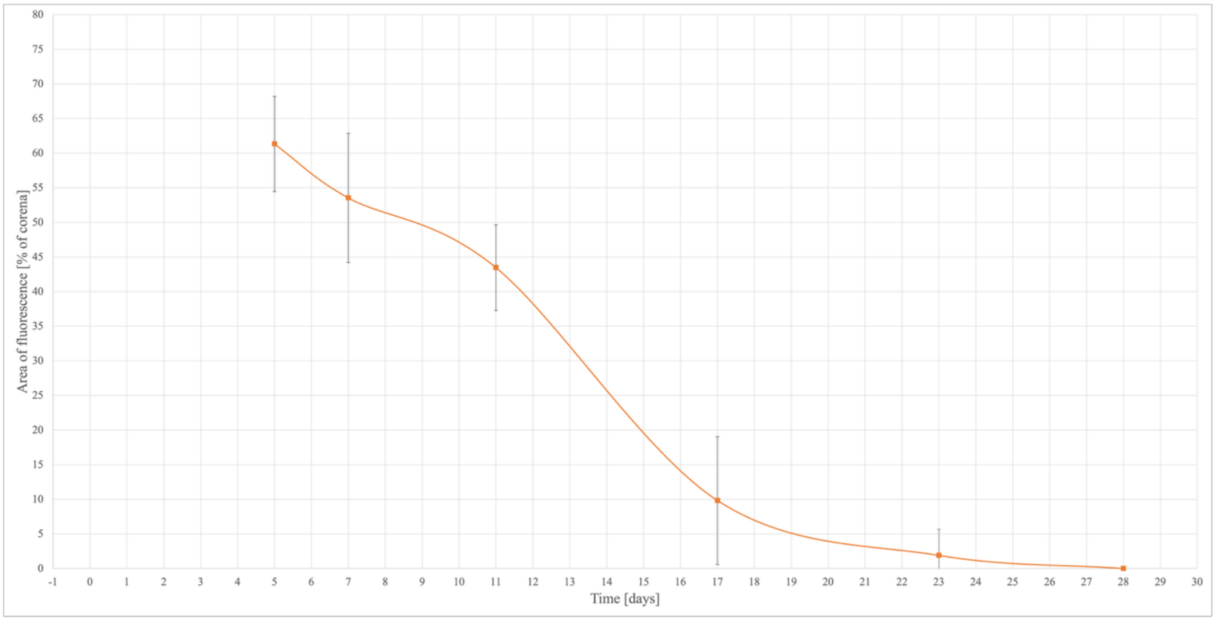

Supplement: Supplementary file 1 — Supplementary Material 1 [file 41598_2025_32842_MOESM1_ESM.pdf]
