# Supplementary material for: Multimodal objective assessment of a porcine limbal stem cell deficiency model for corneal therapy research
Source: Sci Rep. 2025 Dec 20;16:2982. doi: 10.1038/s41598-025-32842-w (PMC12830607; doi:10.1038/s41598-025-32842-w)

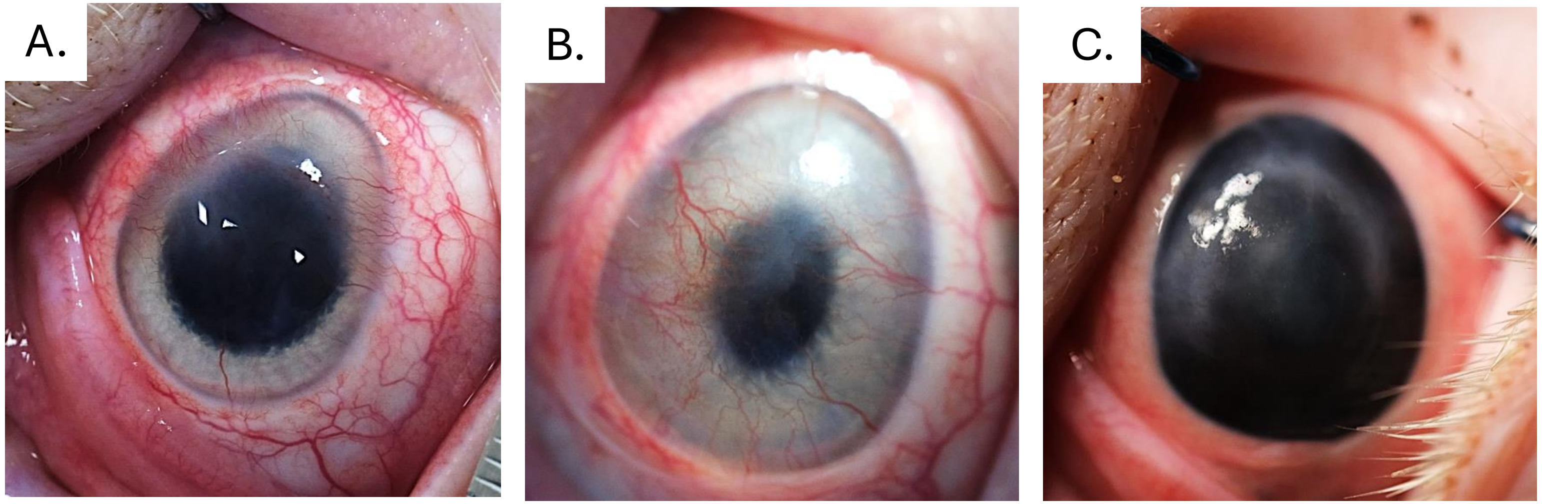

Figure S2. Variability and technical limitations in assessing neovascularization in the porcine cornea.

(A) Slit-lamp photograph showing pronounced 360° corneal neovascularization.

(B) Example of asymmetric vascular ingrowth occurring from two poles of the limbal region, illustrating heterogeneity that may not be captured in histological cross-sections.

(C) Slit-lamp image from a pig with a dark iris, where neovascularization is barely distinguishable due to strong background pigmentation, limiting visibility.

(D) Photograph of an excised cornea demonstrating peripheral vascularization visible under ex vivo microscopy.

(E) Another example of peripheral vessels obscured by glare and light reflections, which frequently interfered with reproducible quantification and prevented reliable standardization of imaging conditions.

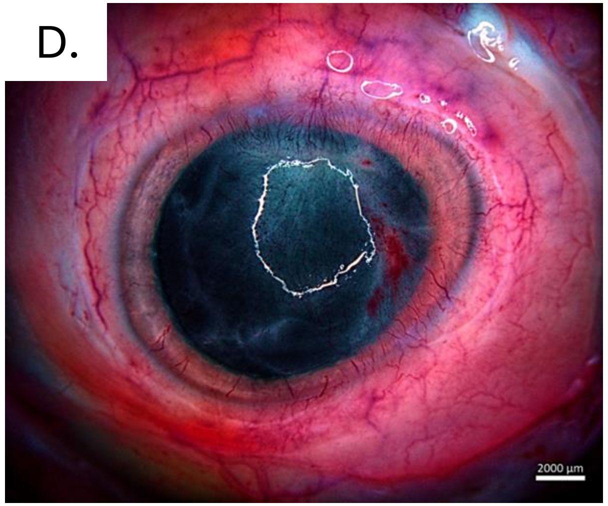

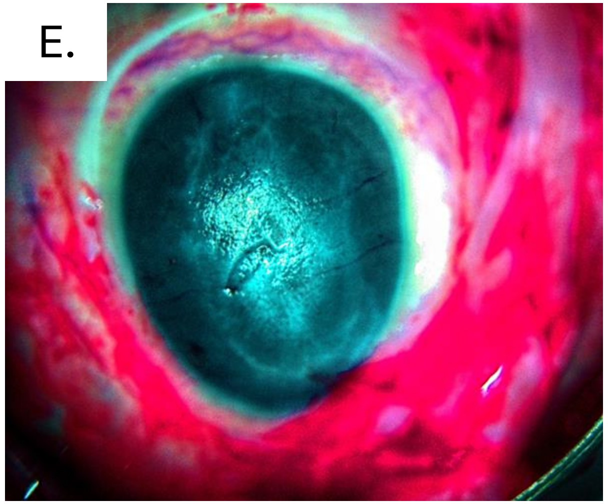

Supplement: Supplementary file 2 — Supplementary Material 2 [file 41598_2025_32842_MOESM2_ESM.pdf]
