# Supplementary material for: Multimodal objective assessment of a porcine limbal stem cell deficiency model for corneal therapy research
Source: Sci Rep. 2025 Dec 20;16:2982. doi: 10.1038/s41598-025-32842-w (PMC12830607; doi:10.1038/s41598-025-32842-w)
Supplement: Supplementary file 3 — Supplementary Material 3 [file 41598_2025_32842_MOESM3_ESM.pdf]

ARRIVE Report

1. Study design

For the study 4 pigs were picked (2 males, 2 females). Each pig's left eye was damaged while right eye served as a control.

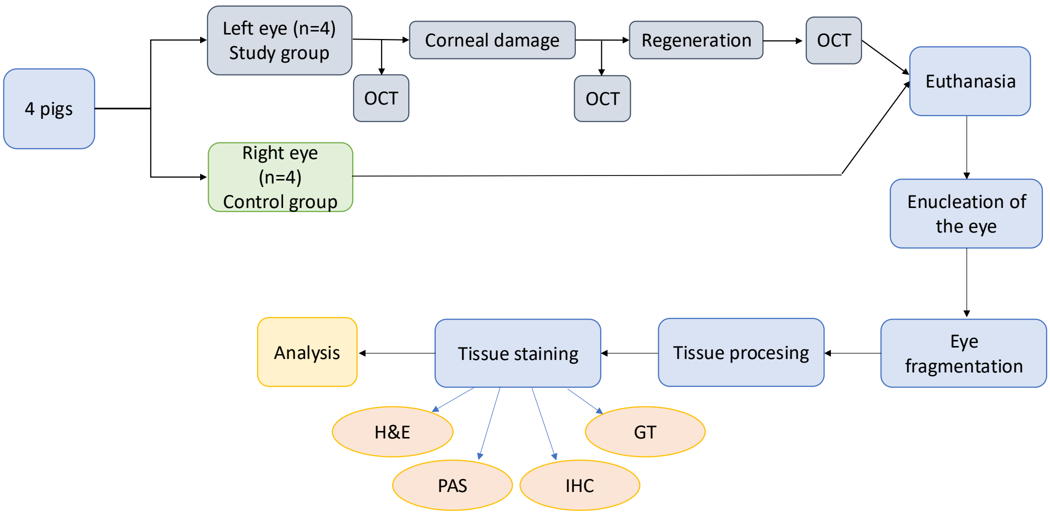

Scheme 1. Study plan presenting most important procedures. OCT- optical coherence tomography. Tissue staining: H&E- hematoxylin and eosine; PAS- periodic acid schiff, IHC- immunohistochemistry, GT- Gomori Trichrome.

2. Inclusion and exclusion criteria

N/A

3. Sample size:

4

4. Randomisation

N/A, each animal served as study group (right eye) and as comparator (left eye)

5. Blinding

No blinding

6. Outcome measures:

Analysis of results:

- corneal thickness
- corneal thickness distribution
- epithelium cells density
- stroma cells density

7. Statistical methods

Statistical analysis was performed with Python program using paired T- test. The level of significance was set at $p<0,05$. Results show differences between LSCD group and control.

8. Results

In right eyes we successfully induced limbal stem cell deficiency. Results have been fully described in main text.

9. Experimental animals

Four individuals (n=4, 2 males, 2 females) of *Sus scrofa domesticus* were used in the experiment. Age was not determined, only weight: Minimal weight at the beginning of the experiment was 20-40kg.

Nr	Species	Sex	Weight (on the procedure day)	Source
20	*Sus scrofa domesticus*	M	28 kg	PL042681623001
21	*Sus scrofa domesticus*	F	31 kg	PL042681623001
22	*Sus scrofa domesticus*	F	53 kg	PL042681623001
23	*Sus scrofa domesticus*	M	63 kg	PL042681623001

10. Experimental procedures:

Experimental procedures were summarized at Scheme 2.

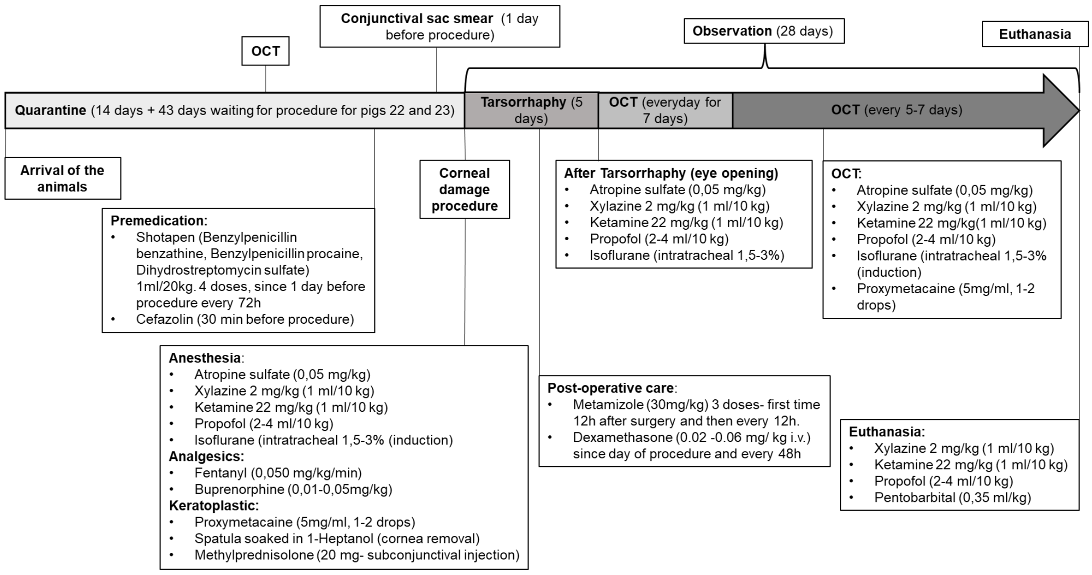

Scheme 2. Procedures performed and medication applied before and during the experiment. All experimental procedures were performed by qualified staff including veterinarian, zootechnician, ophthalmic surgeon.

Pig	Start of the procedure	Monitoring						End of the procedure
**20**	15:10	**Time**	15:40	15:48				15:48
		**Pulse**	96	95				
		**Saturation**	99	95				
		**Blood pressure**						
		**Rectal temperature**	38,4 ˚C	38,3˚C				
**21**	16:00	**Time**	16:30					16:30
		**Pulse**						
		**Saturation**	98					
		**Blood pressure**	85					
		**Rectal temperature**	37,3					
**22**	12:30	**Time**	12:30	12:45	13:00	13:15	13:30	13:40
		**Pulse**	130	121	115	115	110	
		**Saturation**	98	99	100	98	99	
		**Blood pressure**						
		**Rectal temperature**	37,7	38	38,2	38,2	38,2	
**23**	14:00	**Time**	14:15	14:30	14:45			14:45
		**Pulse**	107	105	100			
		**Saturation**	99	100	96			
		**Blood pressure**						
		**Rectal temperature**	37,9	38,3	38,8			

Tab. 2. Animal monitoring during the corneal damage procedure

Conjunctival sac smear- painless procedure, performed by veterinarian using sterile swab stick. Material was used for microbiological culture. Results = no infections
Pigs' preparation: From 12 to 24h before operation animals weren't feed, also no supplements were given to them. Before procedure animals were washed and cleaned to prevent any contamination from the pigs' skin.

11. Housing and husbandry

The pig enclosures were not contain animals of any other species and there was no possibility of the odors of other animals entering. Animals were provided with a diet consisting of an industrial food mixture once a day and had free access to water. To ensure the social needs of the animals, it was planned to keep 2,3 individuals/pen.
Environmental enrichment (rubber toys, balls) was present in the animal pens. The animals have constant access to water, with no restrictions on the amount. Standard feed will be given ad libitum. On the day of treatment procedures, animals will remain fasting.
As required, the animals' living areas will be washed and disinfected to maintain proper zoohygienic conditions. All diagnostic and treatment procedures will be performed in a separate room, out of sight of the animals. Qualified technical personnel will ensure daily monitoring of animal health and welfare. As required, the health status of the animals will be inspected by the Veterinarian. A separate quarantine room is provided for sick animals, and a separate room with pens and equipment for monitoring vital parameters is provided for animals under full anesthesia.
Temperature: 22ºC
Humidity: 55±10%
Light cycle: artificial lighting of 350-500 lux at 1 m height, with 12/12 photoperiod control.
Number of air changes per hour: 16 air changes/hour
