# Supplementary material for: Multimodal objective assessment of a porcine limbal stem cell deficiency model for corneal therapy research
Source: Sci Rep. 2025 Dec 20;16:2982. doi: 10.1038/s41598-025-32842-w (PMC12830607; doi:10.1038/s41598-025-32842-w)
Supplement: Supplementary file 5 — Supplementary Material 5 [file 41598_2025_32842_MOESM5_ESM.pdf]

```python
import pandas as pd
import matplotlib.pyplot as plt
import os

# Update matplotlib font sizes globally
plt.rcParams.update({
    'font.size': 14,           # General font size
    'axes.titlesize': 20,      # Title font size
    'axes.labelsize': 16,      # Axis label font size
    'xtick.labelsize': 14,     # X-axis tick labels
    'ytick.labelsize': 14,     # Y-axis tick labels
    'legend.fontsize': 14,     # Legend font size
    'figure.titlesize': 22     # Figure title font size
})

# Create the folder "corneal thickness profiles" if it doesn't exist
output_folder = "corneal_thickness_profiles"
if not os.path.exists(output_folder):
    os.makedirs(output_folder)

# Load the CSV file
file_path = 'digital_pathology_data.csv'
data = pd.read_csv(file_path)

# Filter rows where 'Classification' is 'Epithelial thickness'
thickness_data = data[data['Classification'] == 'Epithelial thickness'].copy()

# Extract pig number (first two digits from 'Image')
thickness_data.loc[:, 'Pig'] = thickness_data['Image'].str[:2]

# Function to standardize thickness to z-scores for the whole pig
def standardize_thickness_pig(df):
    pig_means = df.groupby('Pig')['Length µm'].mean()
    pig_stds = df.groupby('Pig')['Length µm'].std()

    df.loc[:, 'Z-Score'] = df.apply(lambda row: (row['Length µm'] - pig_means[row['Pig']]) / pig_stds[row['Pig']], axis=1)
    return df

# Apply the standardization
thickness_data = standardize_thickness_pig(thickness_data)

# Separate data for right and left eyes
right_eye_data = thickness_data[thickness_data['Image'].str.contains('P')]
left_eye_data = thickness_data[thickness_data['Image'].str.contains('L')]

# Separate A and B slides for each eye
right_eye_A_data = right_eye_data[right_eye_data['Image'].str.contains('A')]
right_eye_B_data = right_eye_data[right_eye_data['Image'].str.contains('B')]
left_eye_A_data = left_eye_data[left_eye_data['Image'].str.contains('A')]
left_eye_B_data = left_eye_data[left_eye_data['Image'].str.contains('B')]

# Function to smooth the data using a moving average
def smooth_data(y_values, window_size=5):
    return y_values.rolling(window=window_size, min_periods=1, center=True).mean()

# Get the list of unique pigs
pigs = thickness_data['Pig'].unique()

# Set the y-axis scale to (-2.5, 5.5)
y_limits = (-2.5, 5.5)

# Generate separate images for each pig
for pig in pigs:
    for eye, color, slide_data in zip(
        [right_eye_A_data, right_eye_B_data, left_eye_A_data, left_eye_B_data],
        ['red', 'red', 'blue', 'blue'],
        ['Right Eye A', 'Right Eye B', 'Left Eye A', 'Left Eye B']
    ):
        pig_data = eye[eye['Pig'] == pig].sort_values(by='Centroid X µm').copy()
        pig_data.loc[:, 'X'] = range(0, len(pig_data) * 10, 10)
        pig_data.loc[:, 'Z-Score Smoothed'] = smooth_data(pig_data['Z-Score'])

        plt.figure(figsize=(10, 5))
        plt.plot(pig_data['X'], pig_data['Z-Score Smoothed'], color=color, linewidth=2)
        plt.title(f'Pig {pig} - {slide_data} Corneal Epithelium Thickness Profile')
        plt.xlabel('Position on Cornea (µm)')
        plt.ylabel('Thickness (Z-Score)')
        plt.ylim(y_limits)
        plt.grid(True)
        plt.tight_layout()
        plt.savefig(os.path.join(output_folder, f'pig_{pig}_{slide_data.lower().replace(" ", "_")}.png'))
```

```python
        plt.close()

# Generate combined chart
fig, axes = plt.subplots(len(pigs), 4, figsize=(20, len(pigs) * 3), sharex=True, sharey=True)

for i, pig in enumerate(pigs):
    for j, (eye, color, slide_label) in enumerate([
        (left_eye_A_data, 'blue', 'Left Eye A'),
        (left_eye_B_data, 'blue', 'Left Eye B'),
        (right_eye_A_data, 'red', 'Right Eye A'),
        (right_eye_B_data, 'red', 'Right Eye B')
    ]):
        pig_data = eye[eye['Pig'] == pig].sort_values(by='Centroid X µm').copy()
        pig_data.loc[:, 'X'] = range(0, len(pig_data) * 10, 10)
        pig_data.loc[:, 'Z-Score Smoothed'] = smooth_data(pig_data['Z-Score'])

        axes[i, j].plot(pig_data['X'], pig_data['Z-Score Smoothed'], color=color, linewidth=2)
        axes[i, j].set_title(f'Pig {pig} - {slide_label}')
        axes[i, j].set_ylim(y_limits)
        axes[i, j].grid(True)

# Add axis labels
for ax in axes[-1, :]:
    ax.set_xlabel('Position on Cornea (µm)')
for ax in axes[:, 0]:
    ax.set_ylabel('Thickness (Z-Score)')

plt.tight_layout()
plt.savefig(os.path.join(output_folder, 'combined_cornea_thickness_profiles_A_B_standardized_by_pig.png'))
plt.close()

print(f"Charts saved with larger fonts to folder: {output_folder}")
```
